# New approach to environmental investigation of an explosive legionnaires´ disease outbreak in Spain: early identification of potential risk sources by rapid *Legionella* spp immunosensing technique

**DOI:** 10.1186/s12879-018-3605-8

**Published:** 2018-12-27

**Authors:** Fernando Cebrián, Juan C. Montero, Pedro J. Fernández

**Affiliations:** Ministry of Health of the Council of Communities of Castilla - La Mancha, Av. de Madrid s/n, 45600 Talavera de la Reina, Toledo, Spain

**Keywords:** Legionella, Manzanares, Outbreak, Environmental testing, Early detection

## Abstract

**Background:**

An explosive outbreak of Legionnaires’ disease (LD) was identified on 11 December 2015 in Manzanares, Ciudad Real, Spain, and was declared closed by 03 February 2016. The number of declared cases was 593 with 277 confirmed cases so that it can be considered as one of the outbreaks with highest attack rate. This rate could be attributed to the ageing of the population, among others, in addition to various risk factors and habits, and the meteorological conditions (thermal inversion) maintained in this municipality at the time. The Public Health Services succeeded in breaking the bacterial transmission. Several facilities were early identified by microbiological analysis, including a cooling tower and a decorative fountain, as possible infectious sources. Rapid analytical techniques for rapid *Legionella* detection and the shutdown and preventative closure of positive installations have been considered key elements in the control of this outbreak.

**Results:**

Rapid microbiological analysis helped to the early identification of potential risk sources in a Legionnaires´ disease outbreak, reducing decision-making processes according to the actual needs of the intervention in public health and shortening the exposure of the population.

**Conclusions:**

Protocolized and immediate intervention in an outbreak is a crucial issue to reduce their effects on public health. For this, identification and control of the suspicious sources able to disseminate the bacteria and cause the illness is required. Rapid analytical techniques like immunomagnetic separation (IMS) method based on the whole bacterial cell detection are shown as excellent tools to investigate all the potential sources of risk.

## Background

Legionnaires’ disease (LD) is a form of bacterial pneumonia caused by *Legionella* species. While *Legionella pneumophila* is the most frequently isolated species, other *Legionella* species have been documented as causal agent [1].

From environmental habitats, such as surface waters, *Legionella* colonizes the infrastructure of water supply networks, where they proliferate under favourable growth conditions and warm temperatures (35 °C–49 °C) [2–4]. Biofilms are promoted by a poor state, inappropriate design, or the absence of proper maintenance of these systems, providing nutrients for *Legionella*, including organic matter, sludge, corrosion materials and protozoa. These systems can act as transmission devices becoming potential sources of infection when they are contaminated and produce aerosols. Contaminated water droplets, containing the bacteria, remain in the air and can be inhaled and deposited in the human lungs. The accepted form of transmission is the inhalation of water aerosols but aspiration has also been suggested as a way of contracting the disease [5, 6]. Several human risk factors and lifestyle habits can lead to this illness, which is presented as isolated cases or outbreaks in both nosocomial and community-acquired pneumonia scenarios [7].

Infections caused by *Legionella* spp. are considered an emerging public health problem and LD outbreaks are often associated with high mortality rates [8]. The difficulty in investigation of an LD outbreak depends on the number and variety of variables involved. Investigation raises several challenges involving a wide range of installations, which are susceptible to become sources of spreading the bacterium, by itself or in combination. LD must be considered as an emerging disease related to technical progress. Their appearance as a health issue is connected with the introduction of modern industrial processes, air-conditioning and various hot water systems, among others. In addition, number, variety, virulence, persistence, and resistance to disinfection of the bacterial strains involved in the disease must be considered. Recently, evidence on the infection of patients by more than one *Legionella* variant has been reported due either to the co-infection from the same environmental source or the independent infections in a very short period of time [9]. Another variable to be considered is the weather, because meteorological factors (temperature, relative humidity, vapour pressure, among others) have been associated with a higher risk of community-acquired LD and the extent of the outbreak [10, 11]. That could be relevant to understand the survival of *Legionella* in water droplets and the persistence of contaminated aerosols.

In this context, it is unsurprising that no source could be identified in four of the ten largest Legionnaires’ disease clusters in Europe and that no source of legionellae organisms could be found in 57% of environmental investigations [12].

The culture method has been traditionally used to detect *Legionella* in a microbiological investigation. This method facilitates the characterization of the bacterial genome by sequence analysis and, if applicable, it allows establishing the relationship between strains isolated from environmental sources and those isolated from affected patients. Despite its advantages, either the overgrowth of accompanying organisms or the transition of *Legionella* cells into a viable but nonculturable state (VBNC) could compromise the culture results. In this VBNC state, cells are unable to form colonies on standard medium but they remain alive and infective [13, 14]. Other factors that could constrain the culture results are the loss of viability of bacteria after collection and during the sampling, the use of antibiotics in the medium, and the low concentration of legionellae organisms in the samples [15]. All these factors are potential sources of worrying negative results despite the presence of *Legionella*. Finally, the time of obtaining a result (10–12 days) makes it difficult a reliable and timely monitoring of the risk.

In view of the above, a new approach to *Legionella* spp. monitoring by using rapid detection techniques could support the early control of suspicious focus in the first hours of an outbreak. This could help to avoid unnecessary exposure of the citizens to the *Legionella* bacterium [16].

This paper specifically describes the environmental and analytical investigation made within the global investigation of an explosive outbreak occurred in December 2015 in Manzanares (Ciudad Real, Spain), on the basis of the information available in the report prepared by General Directorate of Public Health and Consumer Affairs of the Castilla-La Mancha Community Council [17]. The approach used has focused on shortening the time needed to control the outbreak. It was decided to use a rapid method based on the immunomagnetic separation technique (IMS) and enzyme-immunoassay [18] since a general improvement of the recovery and detectability of *Legionella* spp. in environmental matrices was found in previous work [19].

From our knowledge, the use of rapid techniques to quantifying *Legionella* in environmental samples provides evidence of their usefulness in the rapid intervention in cases of outbreak, as a key and precise tool for the early identification of potential sources of risk.

## Methods

### Environmental samples

The day the outbreak was declared, on Friday 11 December 2015, the elaboration of a directory of installations, susceptible to be classified as being at risk of dispersion and a proliferation of *Legionella*, was essential. First samples were taken at 08 a.m. on 12 December, due to difficulty to work in darkness on the night of 11 December 2015 and continued on December 14 and 15. These samples proceeded from cooling towers and an evaporative condenser. Taking into account the typical dirtiness of this kind of samples and its limited processing, particular with regard to the difficulty of filtration, subsequent sampling alternated different types of installations to prevent the saturation of the laboratory. Regularly water sampling was conducted until the end of the outbreak. In the course of the outbreak, 116 establishments were visited, 68 of these having 30 risk installations in operation. Simultaneously, meteorological information of the municipality was obtained from the database of the State Meteorology Agency (AEMET) in Spain, during the previous days and during the outbreak.

As a general rule, instructions on the sampling procedure were sent by General Directorate of Public Health to district inspectors. The sampling began in the Industrial Park of the town of Manzanares, because possible link between some cases and installations of this Park was observed.

Water samples, preferably with sediments, were taken in duplicate at different points of the risk facilities, in sterile 2 l- containers (total of 4 l). Collected samples were then transported to the Laboratory of the Health Sciences Institute of the Health Council of the Castilla-La Mancha Community Council (Talavera de la Reina, Toledo, Spain). These samples were analyzed by two different techniques:

- A rapid method based on the immunomagnetic separation technique (IMS) and enzyme-immunoassay [18]. The used IMS method, certified by the Research Institute of the Association of Official Analytical Chemist (AOAC-RI), is focused on the 1 h detection of whole cell and designed to promote the bacterial capture attending cell envelop integrity by immune-magnetic particles. This particle-bacteria binding is due to a ligand (antibodies) immobilized on the surface of the particle that binds to antigens expressed on the surface of *Legionella* cells. For this reason this interaction depends on the integrity of the cell envelope and it is independent of the growth ability of cell, often limited in the wild *Legionella*.
  This test has already been evaluated by Public Health laboratories by comparing this test method with q-PCR and conventional culture [19]. Briefly, captured cells can be separated using a magnet, allowing their labelling with an antibody conjugated to an enzyme. They can be resuspended in a medium of constant composition to develop a colour reaction which depends on the quantity of immobilized and labelled *Legionella*. The results are reported as equivalent colony forming units (CFU_eq_).
- Conventional culture method (ISO11731). The culture method is based on the *Legionella* growth on a plate with a selective medium (GVPC agar, Oxoid, Madrid, Spain). The sample concentrate is previously divided into three portions, one of which is acid-washed (pH 2.2, 5 min), other is heat-treated (50 °C, 30 min) and the other is seeded without treatment. The grown colonies with morphology compatible with *Legionella* are confirmed and identified by biochemical, microscopic and serological tests. Cells remain alive for further studies of identification and genomic sequencing. Results, obtained in 10–12 days, are expressed as colony forming units (CFU).
  Additional culture testing based on Annex J of the Draft ISO/DIS 11731 was conducted when confluent or invasive microbial growth was observed on the plates, due to their epidemiological relevance to identify the strain. For this, the other 2 l of the collected samples were used. The isolated strains were identified as *Legionella pneumophila* or non-pneumophila species. Speciation or serogrouping (*Legionella pneumophila* serogroups 1 and 2–15) of isolates was done by using latex agglutination (Microkit, Spain). The identified strains were sent to National Center of Microbiology (Majadahonda, Madrid, Spain) for both phenotypic identification, according to the International Panel of Monoclonal Antibodies, and genomic identification by amplified fragment length polymorphism.

### Clinical samples

A number of 323 clinical samples were sent to the National Centre of Microbiology (most of which were sputum as well as bronchial suction and cultured strains) and 26 of them, mainly sputum, were sent to Laboratory of Genetic of the University of Valencia (Valencia, Spain).

## Results

### The outbreak

On Friday 11 December, the protocol on proceedings in the event of community outbreak of Legionnaires’ disease was activated in the municipality of Manzanares (Ciudad Real, Castilla-La Mancha, Spain), with 4 declared cases having space-time association.

The number of daily cases, according to date of first symptoms, grew in the following days, reaching a maximum of 75 cases on 17 December 2015. Afterwards the number of cases decreased until 25 December 2015. From this date, just a few isolated cases were declared.

The cases were defined by adapting the 2012 EU/EEA case definition [20]. Case definition was based on the following criteria: i) Living in or visiting Manzanares in the 10 days preceding the development of community-acquired pneumonia (CAP) (epidemiological criterion), ii) Pneumonia confirmed by radiology (clinical criterion), iii) A positive result in at least one of the following assays (microbiological criterion for confirmed case): culture, antigen in urine, nested Sequence-Based Typing (SBT) PCR (polymerase chain reaction), seroconversion, iv) A PCR (without sequencing) positive result (microbiological criterion for probable case). Among all the cases that met both epidemiological and clinical criteria, those that met microbiological criterion for confirmed case were defined as confirmed cases and those that met microbiological criterion for probable case were defined as probable cases. Cases that did not meet any of the microbiological criteria were defined as suspect cases. Finally, the cases that did not meet epidemiological criterion or were diagnosed with other causal agent were defined as dismissed cases. A total of 593 cases were declared from which 277 were confirmed cases, 247 were suspect cases, 33 were probable cases and 36 were dismissed cases, according to epidemiological, clinical and microbiological criteria in this outbreak. The outbreak was considered closed on Wednesday 03 February.

The attack rate was 14.9/1000 inhabitants, so it could be considered as one of the known outbreaks with a higher attack rate. Interestingly, the case fatality ratio, 1.4% (4/277), is one of the lowest in the outbreaks scientifically described (Table 1).

**Table 1:** Case-fatality ratios for some Legionnaires’disease outbreaks in Spain

Year	City	Province	no. confirmed cases	Fatality (%)
1973	Benidorm	Alicante	89	3.3
1983	Zaragoza	Zaragoza	81	7.4
1988	Barcelona	Barcelona	56	12.5
1991	Almuñecar	Granada	91	2.2
1996	Alcalá de Henares	Madrid	224	4.0
1999–2000	Alcoy	Alicante	177	6.2
2000	Barcelona	Barcelona	54	4.0
2001	Murcia	Murcia	449	1.1
2002	Mataró	Barcelona	151	1.4
2004	Zaragoza	Zaragoza	32	21.9
2005	Vic	Barcelona	55	5.5
2006	Pamplona	Navarra	146	0.0
2010	Madrid	Madrid	47	12.8
2012	Móstoles	Madrid	63	3.2
2014	Sabadell-Ripollet	Barcelona	48	20.8
2015–2016	Manzanares	Ciudad Real	277	1.4

### Clinical data

A total number of 323 clinical specimens were sent to National Centre of Microbiology. These specimens proceed from 311 patients. Of these samples the 51.39% (166/323) were low-quality samples and their cultivation was not possible. The rest of the samples were processed by culture and the 19.11% (30/157) were positive. The 36.96% (112/303) were positive by PCR method.

A total of 68 strains were sequenced, 57 of them fully sequenced and 11 partially sequenced, corresponding to 12 different strains, with 10 new sequence types not listed in the European Study Group for *Legionella* Infections (ESGLI) database. Among them, *Legionella pneumophila* serogroup 1 Pontiac Philadelphia ST899 (6, 10, 14, 10, 39, 3, 20) was identified as the epidemic strain, being present in the 79.41% (54/68) of the human sequenced samples.

### Description of the environmental and microbiological investigation

On Saturday 12 December 2015, according to the procedure described in the Methods Section, 22 samples were taken involving cooling towers and an evaporative condenser, from facilities located at Industrial Park of Manzanares. On Sunday 13 December 2015, the first results were obtained by rapid IMS method in a period ranging 24–36 h after the samples collection. Results were negative and confirmed by culture on 24 December 2015, twelve days later.

The same methodology was applied on Monday 14 December and Tuesday 15 December 2015. Twenty-two samples were collected from ten installations involving four cooling towers, two sprinkler systems, three decorative fountains and a car wash. On Monday 15 December the Laboratory of Health Sciences Institute of the Health Directorate of Castilla-La Mancha (Talavera de la Reina, Castilla-La Mancha, Spain) reported positive results by IMS method for three of these samples. Two of these samples corresponded to a decorative fountain, both in the water jet (7200 CFUeq/L) and in the tank (3300 CFUeq/L). The third positive corresponded to a cooling tower (210 CFUeq/L).

**Fig. 1:**
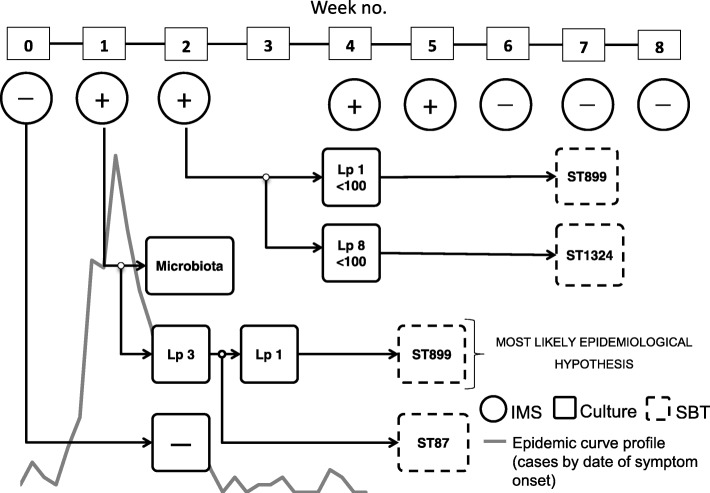
*Legionell*a positivity for each analytical technique (culture, IMS and SBT) along the Legionnaires´ disease outbreak

Initially, the corresponding results for the culture tests were reported as inconclusive on 26 December 2015, with excessive overgrowth of interfering microbiota. The confluent growth on the plates corresponding to the three samples positive by IMS method, appeared two days after plating, revealing the impossibility of *Legionella* isolation. This is way the remaining 2 l were used to try the isolation of some colony, by modifying the culture method according to the guidelines set by the draft ISO/DIS 11731. Despite of this, the colonies isolation remained impossible from the sample of the decorative fountain. However, two colonies of *Legionella pneumophila* were obtained from the sample of the cooling tower: the first one was identified as serogroup 3 (on Wednesday 23 December 2015) and the second one as serogroup 1 (on 28 December 2015). These strains were sent to National Centre of Microbiology to be sequenced using sequenced based typing (SBT) method. The first one was identified as *Legionella pneumophila* serogroup 3 ST87, which did not present any coincidence with any strain isolated in human samples. The second one was identified as *Legionella pneumophila* serogroup 1 subgroup Pontiac Philadelphia ST899, which coincided with the 79% of the strains isolated in human samples (Fig. 1).

Since 15 December 2015, water sampling continued at different risk facilities to further deepen in the outbreak. The Laboratory of the Health Science Institute reported two of these samples as positives, confirming the presence of *Legionella pneumophila* serogroup 1 Pontiac Philadelphia ST899 (in a concentration lower than 100 CFU/L) in the sample of an agricultural irrigation system, located on the outskirts of Manzanares, and *Legionella pneumophila* serogroup 8 ST1324 (in a concentration lower than 100 CFU/L) in the sample of the waste water treatment plant (WWTP) of Manzanares.

Risk facilities with external emissions were submitted to a new screening over the course of January 2016, as preventative measure. Five of the eighty-four tested samples were positive by IMS method: one sample from a cooling tower in an industrial company and two samples from a car wash, all of them without confirmation by culture, and one sample of a cooling tower in a food company with positive culture result for *Legionella* non-pneumophila (in a concentration lower than 100 CFU/L) and finally, a hot sanitary water sample in an old people’s home with positive culture result of *Legionella pneumophila* serogroup 8 ST1324 (in a concentration lower than 100 CFU/L). Moreover, one sample of a car wash which gave negative by IMS method was positive later by culture method detecting *Legionella* non-pneumophila in a concentration lower than 100 CFU/L.

A total of 116 establishments were visited from the beginning until the closure of this outbreak, identifying 68 risk installations, some of them without operation. Thirty installations were more intensively visited, involving inspection or sampling: 12 cooling towers and 1 evaporative condenser, 4 decorative fountains, 3 irrigation systems, and 4 car washes, among others. It is important to highlight that 100% of the cooling towers and evaporative condensers as well as 75% of the decorative fountains were inspected on 15 December 2015, four days after the outbreak declaration. In sum, the Laboratory of Health Sciences Institute of the Health Directorate of Castilla-La Mancha (Talavera de la Reina, Castilla-La Mancha, Spain) analyzed 215 environmental samples, 120 of them processed by culture method (positivity of 5.0%) and 95 processed by IMS method (positivity of 15.83%).

### Meteorological data and weather conditions

**Fig. 2:**
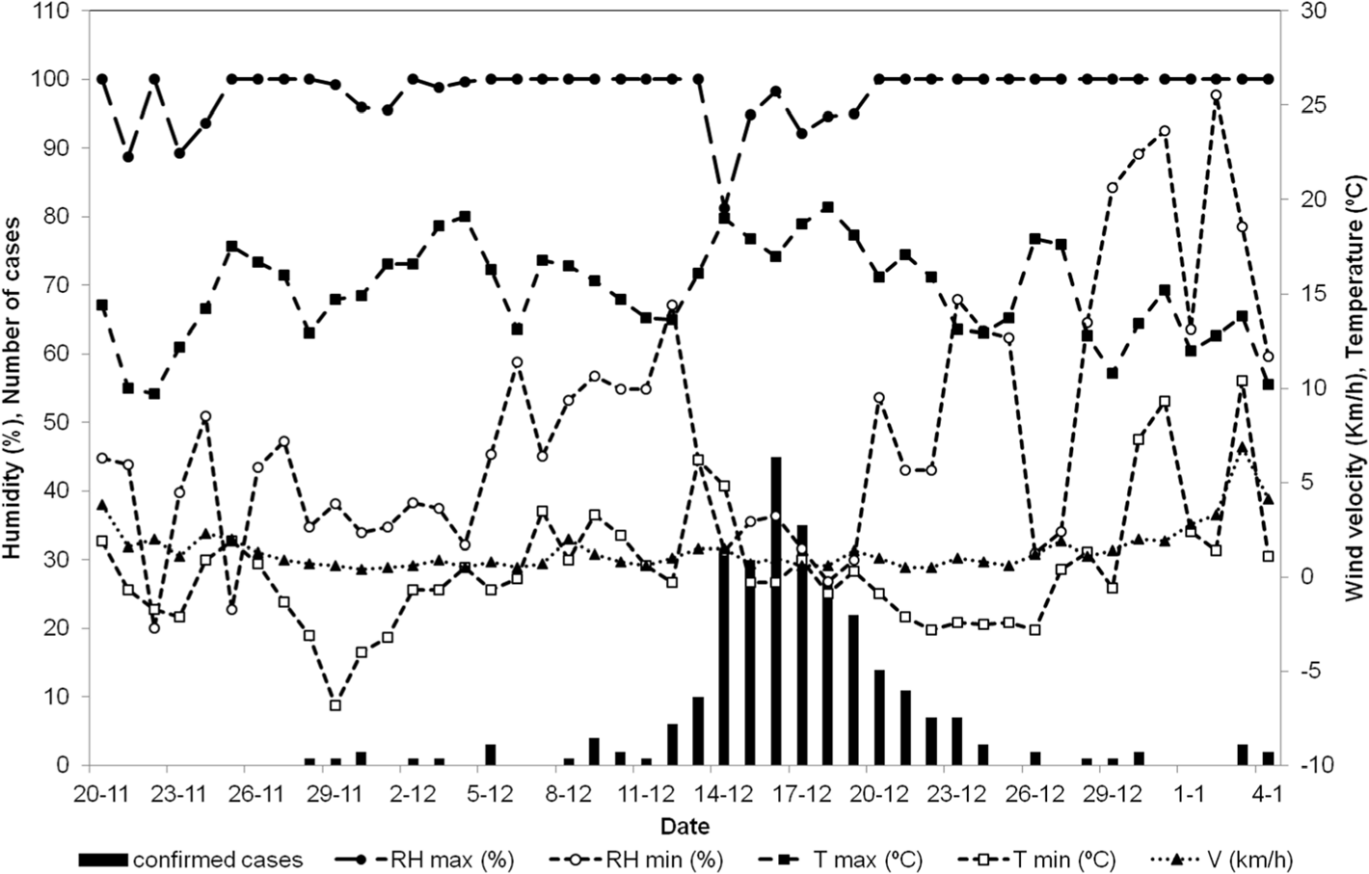
Temporal variations in daily relative humidity (RH, %), daily air temperature (T, ^0^C), wind velocity (V, Km/h) with number of Legionnaires’ disease cases per day (epidemic curve)

Meteorological data showed that between 27 November and 26 December some atmospheric thermal inversion occurred. Wind with a very low average speed (1.8–2.2 km/h) and high humidity (92%) coincided with warmer than usual temperatures and almost without rainfall (Fig. 2).

## Discussion

The identification of the causal focus is crucial for providing answers to questions and requirements associated with an outbreak. However, it should be noted that unequivocal identification of one or several of the installations as responsible of an outbreak depends on the concurrence of many factors. In fact, the source of infection has not been identified in many outbreaks of this kind.

The scenario of the Manzanares outbreak was very complex. Firstly, because a high number of strains were identified in the patients, leaving the possibility open to suspect there could be several installations involved in the outbreak, either direct or indirect (cross-contamination). The wide variety of detected strains may be considered unusual in the context of the current literature. Secondly, because the difficulty in isolating *Legionella* by culture method, particularly in environmental samples, given the dirtiness of the samples taken from installations with poor or absent maintenance. In this regard, no culture results were obtained for the samples taken from a decorative fountain in a bus station, due to presence of interfering microbiota, despite their positivity by rapid IMS method. This becomes of particular importance because epidemiological models that best explain the most of the cases points to this fountain. Thirdly, because the difficulty in knowing the operation of some installations, in particular the cooling tower of a laundry company, where the epidemic strain ST899, corresponding to 79.41% (54/68) of the clinical strains, was identified. However, there is doubt about the partial or total operation of this installation during the exposure period. Fourthly, meteorological conditions would have facilitated the presence of the aerosols in the environment, which could partly explain the high number of cases by inhabitant [21] together with the characteristics of an aging population, concentrated in just one nucleus and having an important industrial park.

From an environmental point of view, a critical issue in a legionnaires’ disease outbreak is to identify the potential sources of risk and to determine causality relating them with the focus of infection. Comparison between environmental and clinical strains allows the confirmation of an installation as origin of the outbreak. The sequenced strains were obtained after their growth on a culture plate, although alternative methods exist.

Furthermore, urgent intervention without falling into improvisations is required in addressing the outbreak. The reason is to achieve the identification of the suspicious focus and their precautionary closure, reducing or minimizing the effects. This intervention is provided by the use of rapid methods of *Legionella* detection, like the IMS method which detects cells with envelope integrity, unlike other rapid techniques like PCR.

**Fig. 3:**
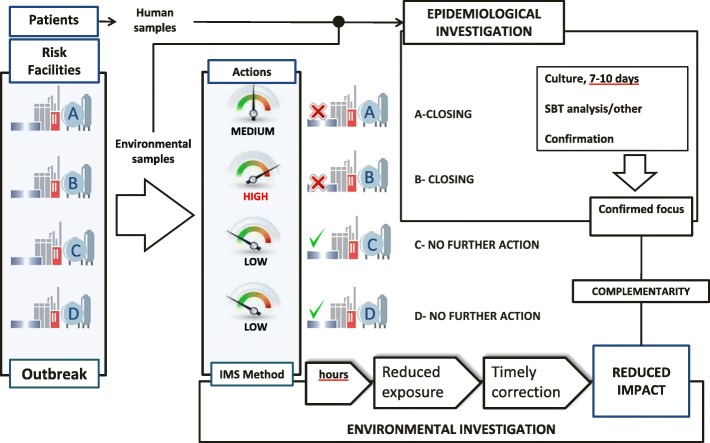
Environmental investigation strategy in connection with microbiological and epidemiological investigation for the Legionnaires´ disease outbreak

In the case of the Manzanares outbreak, this IMS rapid test was considered critical in i) allowing to locate potentially infective sources in a short time, ii) focusing the environmental research and increasing the efficacy of the inspections and sampling, iii) increasing the sample throughput, iv) rerouting the culture testing for the purpose of isolating colonies to be identified and compared to the colonies isolated from patients, and v) supporting the decision making process for the control of the outbreak (Fig. 3). In contrast the separated bacterium is not available for subsequent studies.

Likewise, during sampling procedure, the owners of the facilities were informed of the obligation to stop their operation until the obtaining of analytical results. In the event of a negative result they could continue to function but always after applying water treatment.

In the outbreak scenario, the culture technique should be useful to isolate the epidemic strain, but a 10 days period is considered too long. By using the IMS method, analytical results were obtained in 24–48 h, demonstrating that rapid techniques are useful in outbreak scenarios where immediate response times are required.

## Conclusions

Protocolized and immediate intervention in an outbreak is a crucial issue to reduce their effects on public health. For this, identification and control of the suspicious sources able to disseminate the bacteria and cause the illness is required. Rapid analytical techniques like IMS method based on the whole bacterial cell detection are shown as excellent tools to investigate all the potential sources of risk.
